# Perpendicular Magnetic Anisotropy and Hydrogenation-Induced Magnetic Change of Ta/Pd/CoFeMnSi/MgO/Pd Multilayers

**DOI:** 10.1186/s11671-018-2628-9

**Published:** 2018-07-25

**Authors:** Qing Zhang, Huarui Fu, Caiyin You, Li Ma, Na Tian

**Affiliations:** School of Materials Science and Engineering, Xi’an University of Technology, Xi’an, 710048 People’s Republic of China

**Keywords:** Spin gapless semiconductor, Perpendicular magnetic anisotropy, Hydrogenation-induced magnetic change

## Abstract

The perpendicular magnetic anisotropy (PMA) has been achieved in Ta/Pd/CoFeMnSi (CFMS)/MgO/Pd film, in which the Heusler compound CoFeMnSi is one of the most promising candidates for spin gapless semiconductor (SGS). The strong PMA, with the effective anisotropy constant *K*_eff_ of 5.6 × 10^5^ erg/cm^3^ (5.6 × 10^4^ J/m^3^), can be observed in the Ta/Pd/CFMS (2.3 nm)/MgO (1.3 nm)/Pd films annealed at 300 °C. In addition, it was found that the magnetic properties of Ta/Pd/CFMS/MgO/Pd films are sensitive to hydrogen (H_2_) under a weak magnetic field (< 30 Oe), whose residual magnetization (M_*r*_) decreased from 123.15 to 30.75 emu/cm^3^ in the atmosphere with H_2_ concentration of 5%.

## Background

Nowadays, hydrogen (H_2_) as one of the new clean and efficient energy source has attracted more attentions, and thus, ensuring the safety of its usage becomes more and more significant. Solid state conductometric gas sensor is commonly used to detect the hydrogen, but it lacks the chemical selectivity and humidity sensitivity [1]. Recently, the magnetic sensors have been proved to be a useful way to detect the various gases, especial hydrogen, in which the film structures containing palladium (Pd) layer are currently under the intense study because the Pd possesses the high sensitivity [2] and selectivity [3] to hydrogen. Thus, the Pd-containing films can be used as an effective catalyst for hydrogen dissociation and absorption [4]. To date, many studies have reported the hydrogenation-induced magnetic change in Pd-rich magnetic alloy film and Pd/ferromagnetic layer (Pd/FM) multilayer films, such as Co_17_Pd_83_ [1], Pd/Fe [5], [Co/Pd]_12_ [6], and Pd/Co/Pd [7] films. The hydrogenation-induced magnetic change can be attributed to the swell of Pd lattice due to the hydrogen absorption, which could contribute to about 2–3% volume expansion.

On the other hand, Pd as a noble metal is commonly used for realizing the perpendicular magnetic anisotropy (PMA) owing to the *d*-*d* electron orbital hybridization at the interfaces of Pd/ferromagnetic layer. This critical interfacial effect of electron orbital hybridization is very sensitive to the interfacial strain or stress [8], which could be brought in through the volume evolution of noble metal. Therefore, high sensitivity of hydrogen-induced magnetic change could be expected from the PMA film with Pd layer by making use of the strong interfacial dependences of perpendicular magnetic anisotropy.

So far, a huge number of studies of PMA have been reported, which have been originated from the *d*-*d* or *d*-*p* electron orbital hybridizations of ferromagnetic layer and noble metal (Pt, Pd) or oxygen of oxides at the interfaces [9–12]. Moreover, Heusler quaternary compound CoFeMnSi (CFMS) has been proved to be a spin gapless semiconductor (SGS) [13–15], which is also very sensitive to the external field [16], showing the potential advantages of being a sensor. In this work, the Ta/Pd/CoFeMnSi/MgO/Pd-structured films were designed to achieve the strong PMA by the interfacial effect, and the hydrogenation-induced magnetic change was explored. Different from the above reports [1, 5–7], the perpendicular magnetic anisotropic film structure and SGS-like CoFeMnSi ferromagnetic layer are all sensitive to the extrinsic effects, such as interfacial stress or strain. Thus, the highly sensitive change of magnetism could be expected from the films under a low magnetic field.

## Methods

Four sets of samples were prepared as follows: Ta (6 nm)/Pd (2.4 nm)/CoFeMnSi (2.3 nm)/MgO (*t*_MgO_)/Pd (2 nm) (*t*_MgO_ = 0.9–1.5 nm) (hereinafter refer to Ta/Pd/CFMS (2.3 nm)/MgO (*t*_MgO_)/Pd), Ta (6 nm)/Pd (2.4 nm)/CoFeMnSi (*t*_CFMS_)/MgO (1.3 nm)/Pd (2 nm) (*t*_CFMS_ = 1.9–3.1 nm) (hereinafter refer to Ta/Pd/CFMS (*t*_CFMS_)/MgO (1.3 nm)/Pd), Ta (6 nm)/Pd (2.4 nm)/CoFeMnSi (2.3 nm)/Pd(2 nm) (hereinafter refer to Ta/Pd/CFMS/Pd), and Ta (6 nm)/CoFeMnSi (2.3 nm)/MgO (1.3 nm)/Pd(2 nm) (hereinafter refer to Ta/CFMS/MgO/Pd). All films were deposited on the Si substrate by magnetron sputtering system under a base pressure better than 2.6 × 10^−5^ Pa at room temperature. The purity of CoFeMnSi target was better than 99.9%. The CFMS layer was deposited under Ar pressure of 0.9 Pa with the DC power of 40 W. The MgO layer was deposited under Ar pressure of 0.2 Pa with the RF power of 150 W. The Ta layer was deposited under Ar pressure of 0.3 Pa with the DC power of 50 W, and Pd layer was deposited under Ar pressure of 0.3 Pa with the DC power of 25 W. The films were annealed within the temperature range from 250 to 450 °C for 30 min under a vacuum chamber below 10^−4^ Pa.

The magnetic properties were characterized by vibrating sample magnetometer (VSM: Lakeshore 7404). The electric transport property measurement system (ET Chen, ET9000) was used to monitor the Hall resistivity with the change of hydrogen absorption and desorption in real time. All measurements were carried out at room temperature and atmospheric pressure. The total gas flow rate was fixed at 3.5 L/min for the sensitivity of hydrogen gas. The hydrogen concentration was tuned by controlling the gas flow rate of the mixed gas (H_2_:Ar = 5:95) and nitrogen gas (N_2_).

## Results and Discussion

**Fig. 1:**
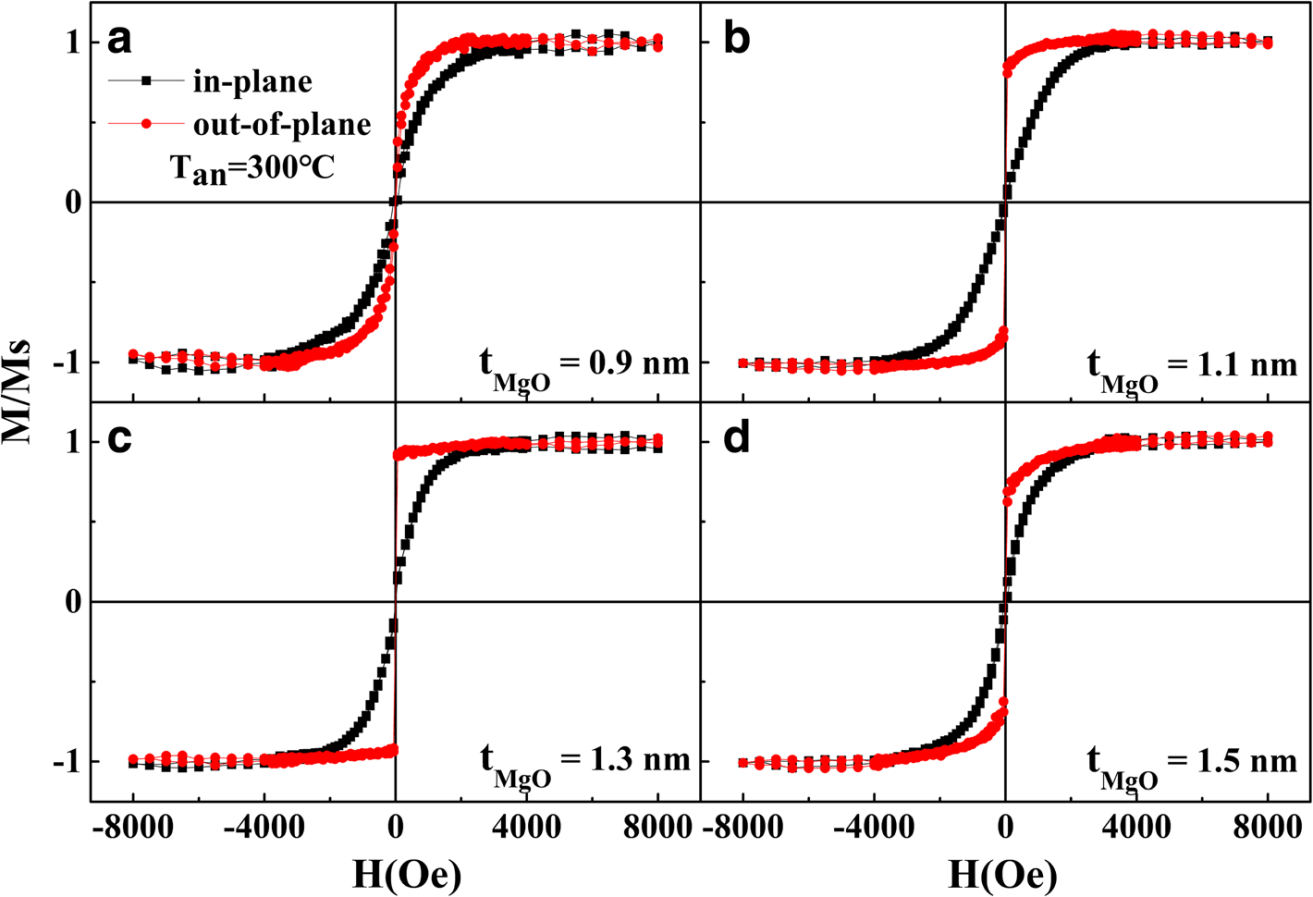
In-plane and out-of-plane M-H loops of Ta/Pd/CFMS (2.3 nm)/MgO (*t*_MgO_)/Pd annealed at 300 °C. **a** *t*_MgO_ = 0.9 nm. **b** *t*_MgO_ = 1.1 nm. **c** *t*_MgO_ = 1.3 nm. **d** *t*_MgO_ = 1.5 nm

To understand the effect of the MgO layer thickness on PMA, Fig. 1 shows magnetic hysteresis loops measured along the in-plane and out-of-plane directions for Ta/Pd/CFMS (2.3 nm)/MgO (*t*_MgO_)/Pd films annealed at 300 °C with varied thicknesses *t*_MgO_. All samples are easily magnetized along the out-of-plane direction, and large saturation fields are needed along the in-plane direction, exhibiting PMA behaviors. The strength of PMA firstly increases with increasing *t*_MgO_ and reaches the maximum value with the squareness (M_*r*_/M_*s*_) close to 1 when *t*_MgO_ = 1.3 nm while obviously decreases with further increasing *t*_*MgO*_.

**Fig. 2:**
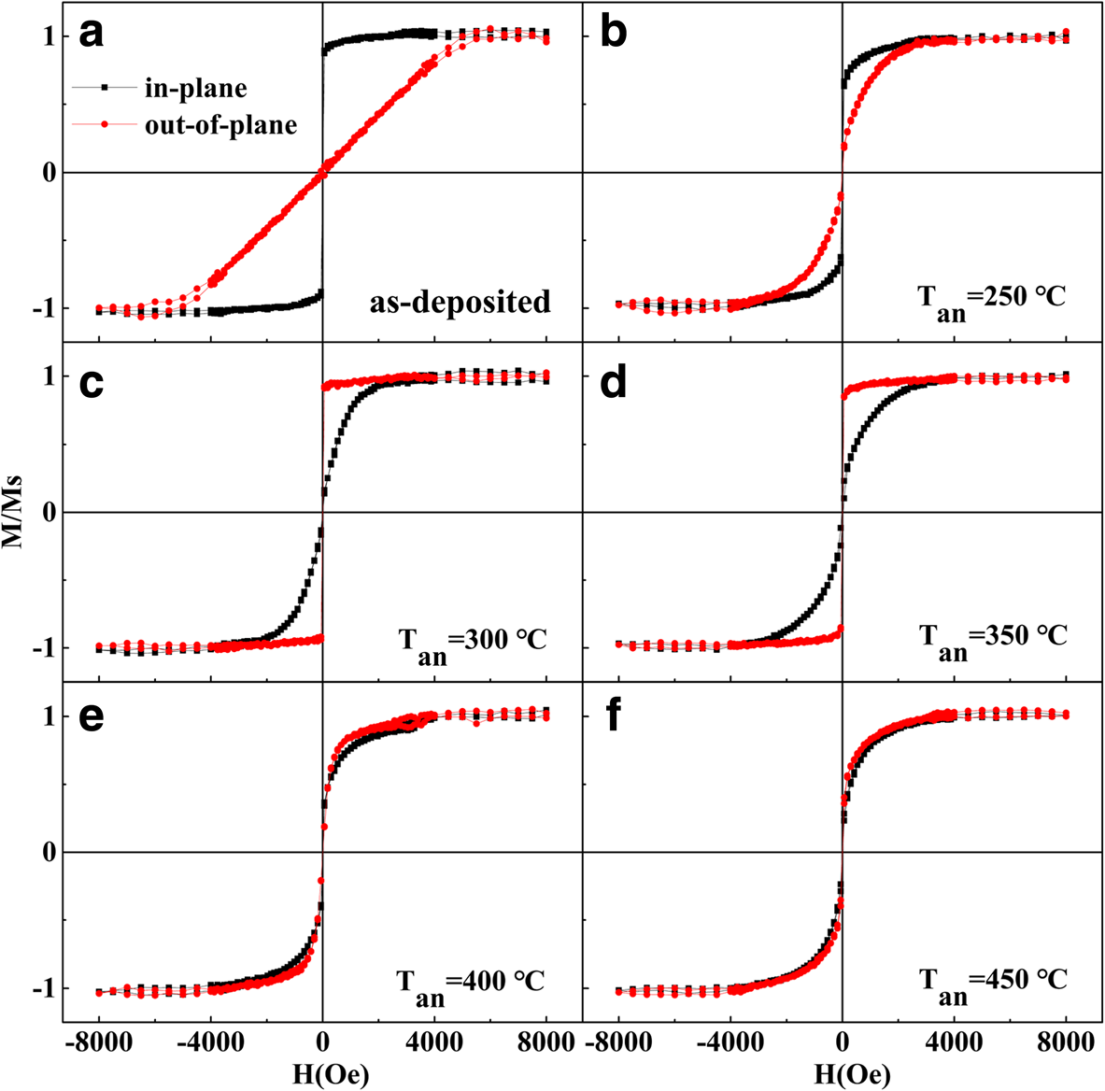
In-plane and out-of-plane M-H loops of the Ta/Pd/CFMS (2.3 nm)/MgO (1.3 nm)/Pd films annealed at different temperatures. **a** As-deposited. **b** 250 °C. **c** 300 °C. **d** 350 °C. **e** 400 °C. **f** 450 °C

In order to elucidate the influence of annealing temperature on PMA, Fig. 2 shows the M-H loops of the Ta/Pd/CFMS (2.3 nm)/MgO (1.3 nm)/Pd films annealed at different temperatures (250–450 °C). The as-deposited sample exhibits an in-plane magnetic anisotropy (IMA) as seen in Fig. 2a. Magnetic anisotropy did not change after annealing at a low temperature of 250 °C (Fig. 2b). The easy magnetization axis of the sample annealed at 300 °C shifted to out-of-plane direction, showing strong PMA (Fig. 2c). The PMA could be maintained after *T*_an_ rising to 350 °C, but the squareness decreased. With further increasing the *T*_an_, the PMA was destroyed and the easy magnetization axis shifted back to in-plane orientation (Fig. 2e, f). The results indicate that the strong PMA can only be achieved at the proper annealing temperature and is easy to be deteriorated at a higher annealing temperature. This is because high annealing temperature could give rise to the intensified inter-diffusion of the atoms at the interface and deteriorate the electron orbital hybridization, which is consistent with our previous reports [9, 12, 17, 18].

**Fig. 3:**
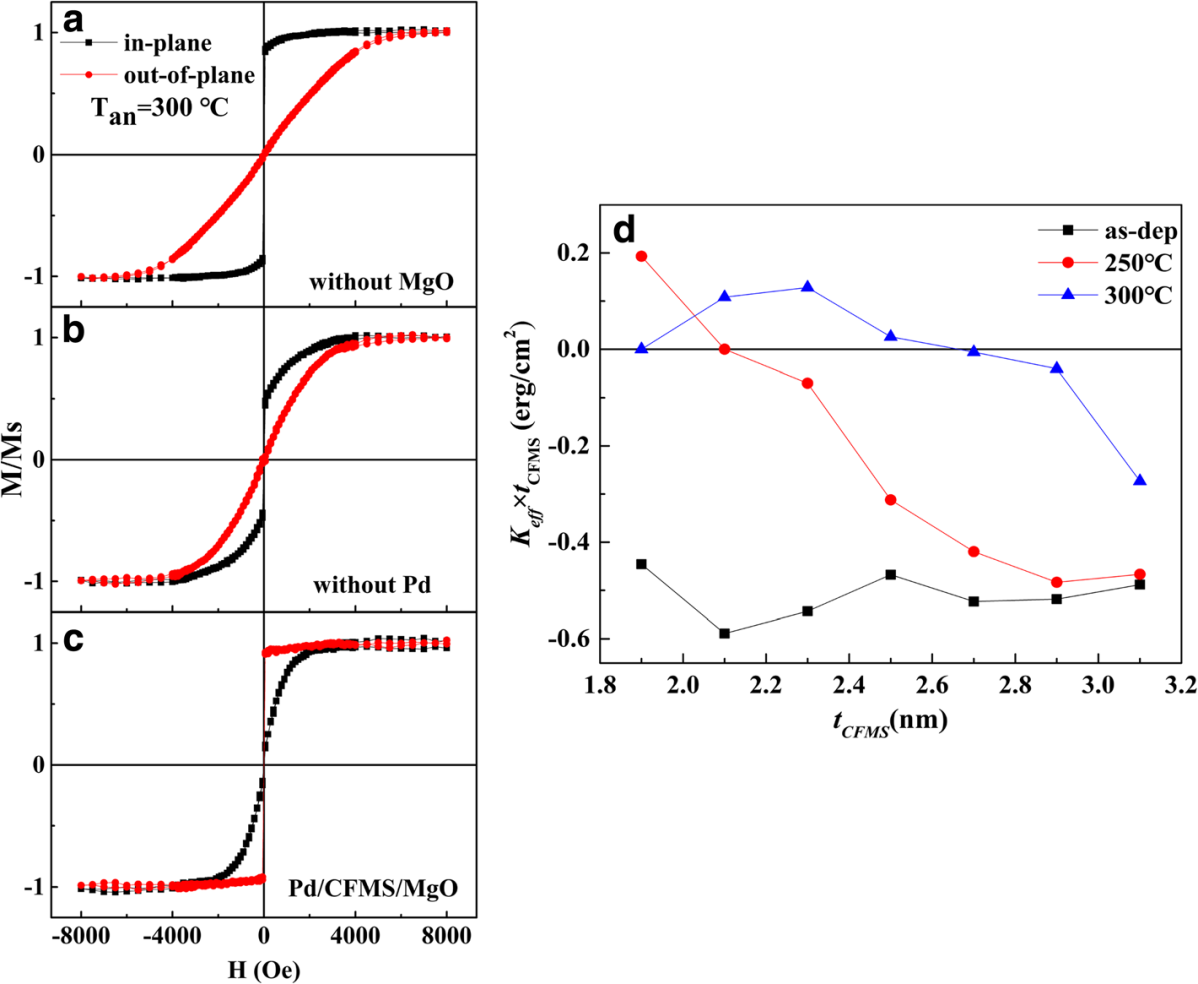
The M-H loops of **a** Ta/Pd/CFMS/Pd, **b** Ta/CFMS/MgO/Pd, and **c** Ta/Pd/CFMS (2.3 nm)/MgO (1.3 nm)/Pd annealed at 300 °C and **d** the CFMS layer thickness dependence of *K*_eff_ × *t*_CFMS_ product for Ta/Pd/CFMS (*t*_CFMS_)/MgO (1.3 nm)/Pd annealed at different temperatures

In order to clarify the interfacial effect on the PMA in the Ta/Pd/CFMS/MgO/Pd films, the M-H loops of different film stacks were given in Fig. 3a–c. As shown in Fig. 3a, the film without MgO layer presents strong IMA behavior. But for the film without the bottom Pd layer, the easy magnetization axis of the Ta/CFMS/MgO/Pd sample exhibits a slight shift from the in-plane direction, showing the weak IMA (Fig. 3b). The strong PMA is observed in the film after inserting the Pd and MgO layers (i.e., Ta/Pd/CFMS/MgO/Ta) as seen in Fig. 3c, implying that both the Pd/CFMS and CFMS/MgO interface are essential for realizing PMA, and the contribution of CFMS/MgO interface to the PMA plays a major role [12, 17]. That is, an appropriate amount of Co-O bonds at the CFMS/MgO interface is helpful for achieving the optimum PMA. The thin MgO layer makes CFMS/MgO underoxidized (Fig. 1a, b), and thick MgO layer makes CFMS/MgO overoxided (Fig. 1d), which both weaken the PMA [11]. As shown in Fig. 1c, the sample with *t*_MgO_ = 1.3 nm has the proper Co-O bonds in the CFMS/MgO interface to obtain strong PMA.

To quantify the PMA strength in the Ta/Pd/CFMS/MgO/Pd films, the effective anisotropy constant *K*_eff_ is given by

1 $$ {K}_{\mathrm{eff}}={K}_v-2\uppi {M}_S^2+{K}_S/{t}_{\mathrm{CFMS}} $$

where *K*_*V*_ and *K*_*S*_ are the bulk and interface anisotropy, respectively. *K*_eff_ is determined by the differences of magnetization energy between the hard and easy magnetization directions. The positive *K*_eff_ represents PMA, and the negative *K*_eff_ represents IMA. The product of *K*_eff_ × *t*_CFMS_ as a function of *t*_CFMS_ for the Ta/Pd/CFMS (*t*_CFMS_)/MgO (1.3 nm)/Pd films annealed at different temperatures is shown in Fig. 3d. All as-deposited films present the negative *K*_eff_, implying the absence of PMA. The PMA of the films annealed at 250 °C can only be observed with *t*_CFMS_ = 1.9 nm. For the films annealed at 300 °C, the PMA can be maintained within a wide *t*_CFMS_ range (below 2.7 nm). The largest *K*_eff_ value of the sample is 5.6 × 10^5^ erg/cm^3^ (5.6 × 10^4^ J/m^3^) with *t*_CFMS_ = 2.3 nm.

**Fig. 4:**
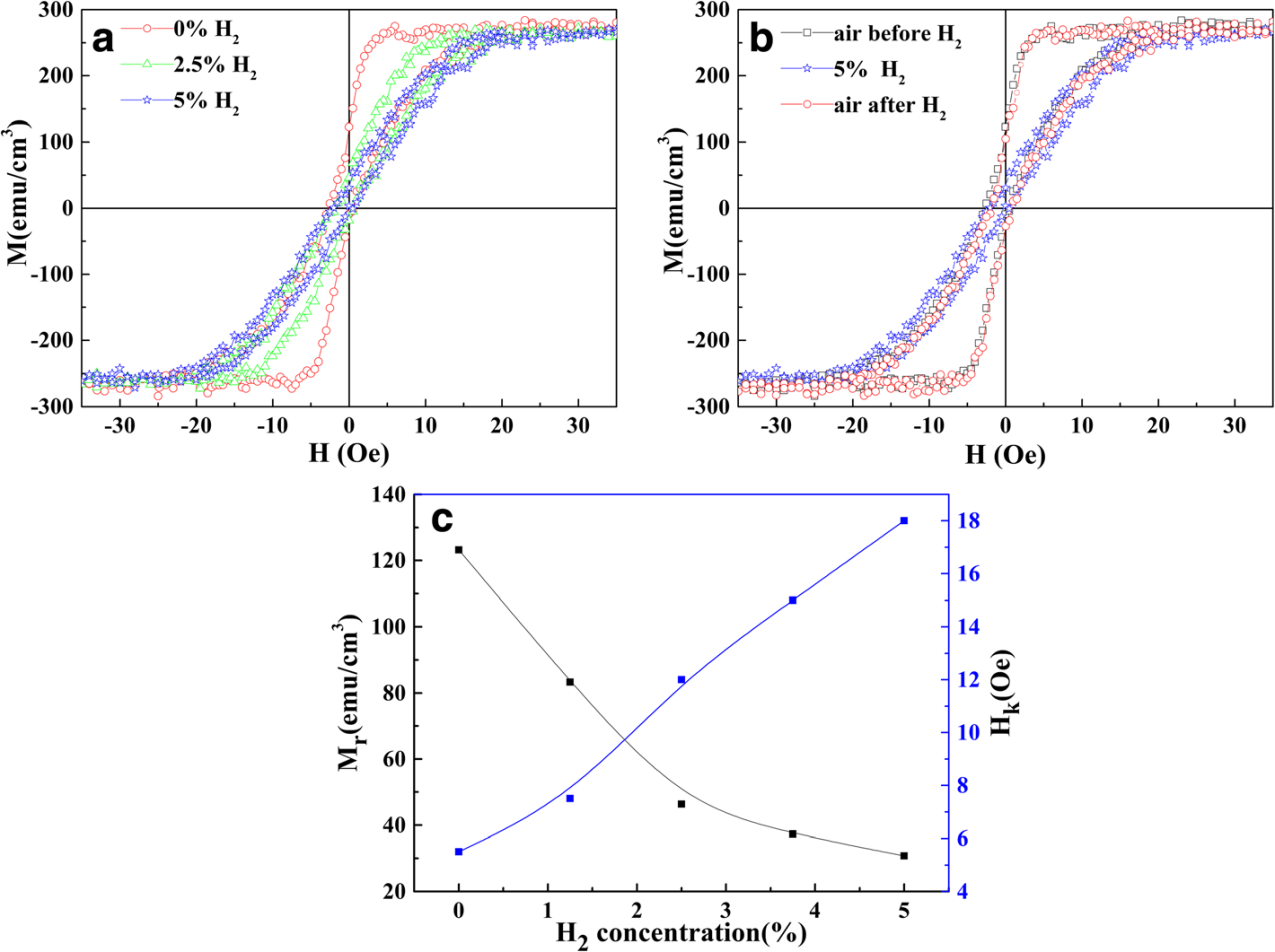
The out-of-plane M-H loops for Ta/Pd/CFMS (2.3 nm)/MgO (1.3 nm)/Pd films annealed at 300 °C. **a** Under H_2_ introduction. **b** Comparison after removing H_2_. **c** The dependence of M_*r*_ and H_*k*_ on H_2_ concentration

As shown above, the PMA is very sensitive to the interfacial environment, which could be also affected through the gas absorption or desorption of noble metal Pd. Thus, the hydrogenation-induced magnetic change was investigated on the Ta/Pd/CFMS (2.3 nm)/MgO (1.3 nm)/Pd films annealed at 300 °C. The M-H loops were checked under different gas atmospheres through varying the H_2_ concentration as shown in Fig. 4a. Noted here, the M-H loops cannot be affected by the pure nitrogen N_2_ and pure argon Ar atmospheres (data did not show here). After introducing H_2_, the M-H loop significantly changes, and the easy magnetization axis shifts away from the out-of-plane direction, showing a large saturation field of the out-of-plane magnetic curve. It is found that the saturation field increases with increasing H_2_ concentration. The sample exhibits an excellent hydrogen sensitivity under a small applied magnetic field (< 30 Oe). Figure 4b shows the M-H loops measured under the air atmosphere before and after the addition of H_2_. It can be seen that the M-H loop is well back to the initial state after removing H_2_. As shown in Fig. 4c, M_*r*_ decreases from 123.15 to 30.75 emu/cm^3^ (decreased 75%), and the saturation field (H_*k*_) increases from 5.5 to 18 Oe with increasing H_2_ concentration from 0 to 5%.

**Fig. 5:**
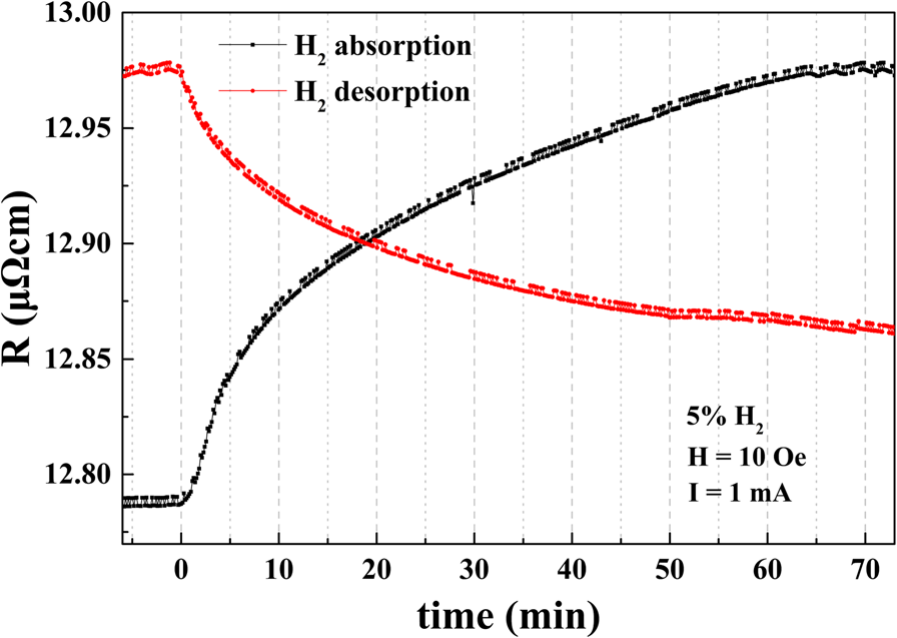
The dependence of the Hall resistivity on time under H_2_ absorption and desorption for Ta/Pd/CFMS (2.3 nm)/MgO (1.3 nm)/Pd films annealed at 300 °C

Figure 5 shows the dependence of the Hall resistivity on time for H_2_ absorption and desorption in Ta/Pd/CFMS (2.3 nm)/MgO (1.3 nm)/Pd annealed at 300 °C. As shown in Fig. 5, H_2_ absorption rate is faster than the desorption rate. The Hall resistivity gradually increased to be saturated in 70 min after being exposed to H_2_. However, through introducing N_2_ to expel H_2_, the Hall resistivity only decreases 60% due to undesorpted H_2_. The Hall resistivity increased/decreased quickly at the beginning (first 10 min) under the processes of H_2_ absorption/desorption, since the hall resistivity is mainly related to the magnetic layer (CoFeMnSi). Thus, it can be deduced that the resistivity changes at the beginning are majorly originated from the interfacial variations between Pd and CoFeMnSi layers due to H_2_ absorption/desorption. The resistivity change at the later stage could be the intrinsic changes of multilayer films due to the absorbed H_2_. In comparison to Fig. 4b, the magnetic detection of multilayered films could be very reproducible owing to the well recovering of the magnetic performance in comparison to the resistivity variations.

As mentioned above, the hydrogenation-induced magnetic change mainly comes from the stress acting on the film with H_2_ absorption of Pd in the Ta/Pd/CFMS (2.3 nm)/MgO (1.3 nm)/Pd film system [19]. It is known that Pd is an effective catalyst for dissociating the hydrogen molecule [4]. Hydrogen molecules are adsorbed and dissociated to hydrogen atoms on the surface of the Pd layer. The lattice of Pd could be expanded with the absorption of hydrogen atoms [20], which in turn has tensile stress to its adjacent MgO and CFMS layer, leading to the controllable magnetism of CoFeMnSi. After discharging H_2_, the hydrogen atoms can escape from the Pd membrane surface [21], causing the recovering of the magnetic performance.

## Conclusions

We demonstrated the strong PMA and the hydrogenation-induced magnetic change in the Ta/Pd/CFMS/MgO/Pd films. The loop squareness (M_*r*_/M_*s*_) is close to 1 for the sample with *t*_CFMS_ = 2.3 nm and *t*_MgO_ = 1.3 nm after annealing at 300 °C, obtaining a high perpendicular magnetic anisotropy *K*_eff_ value of 5.6 × 10^5^ erg/cm^3^. Owing to the hydrogen absorption of Pd, the annealed Ta/Pd/CFMS/MgO/Pd film at 300 °C exhibited excellent hydrogen sensitivity; the residual magnetization (M_*r*_) decreased 75% under the atmosphere with H_2_ of 5%.

## Abbreviations

CFMS: CoFeMnSi
IMA: In-plane magnetic anisotropy
PMA: Perpendicular magnetic anisotropy
